# Supplementary material for: Identification of photoperiod-induced specific miRNAs in the adrenal glands of Sunite sheep (Ovis aries)
Source: Front Vet Sci. 2022 Jul 22;9:888207. doi: 10.3389/fvets.2022.888207 (PMC9354845; doi:10.3389/fvets.2022.888207)
Supplement: Supplementary file 1 [file Data_Sheet_1.ZIP › Supplementary file/miRDeep2 file.zip!/miRDeep2 file/Know_Structure/LP42A3/oar-mir-374b.pdf]

miRBase precursor : oar-mir-374b
Total read count : 27774
oar-miR-374b read count : 27075
remaining reads : 699

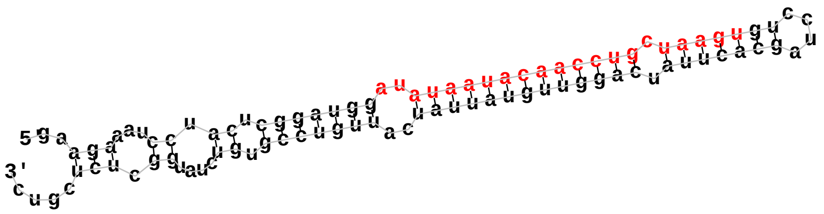

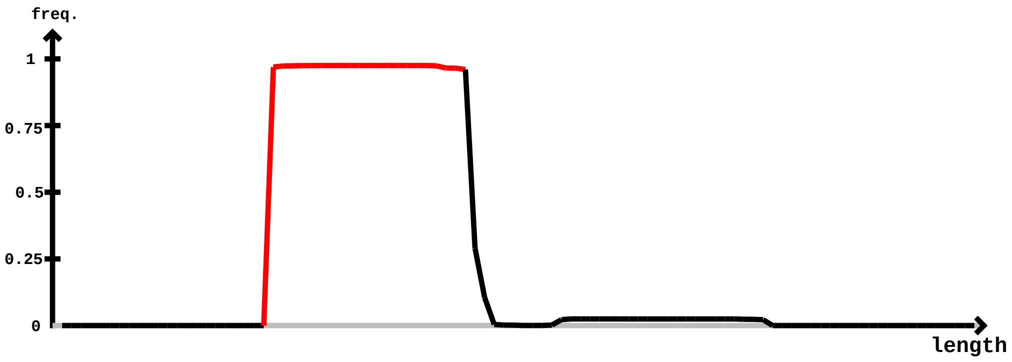

oar-miR-374b

```
5'- gaagaaauccuacucggauggauauaauacaaccugcuaaguguccuagcacuuaucagguuguauuaucauuguccgugucuauggcucucguc   -3'    exp
    ((((..((...((.(((((((..((((((((((((.(((((((....))))))).))))))))))))..))))))).))..))..)).))...    reads      mm         sample
    ....................gauauaauacaaccugcuaagu.....................................................    1          0          seq
    .....................auauaCuacaaccugcu.........................................................    1          1          seq
    .....................auauaauacaaccugcu.........................................................    33         0          seq
    .....................auauaauacaaccugcua........................................................    231        0          seq
    .....................auauaauacaaccugcuaa.......................................................    5          0          seq
    .....................auauaauacaaccugcuaag......................................................    146        0          seq
    .....................auauaauacaaccugcuaagu.....................................................    18584      0          seq
    .....................auauaauacaaccugcuaagug....................................................    5129       0          seq
    .....................auauaauacaaccugcuaagugu...................................................    2736       0          seq
    .....................auauaauacaaccugcuaaguguc..................................................    42         0          seq
    .....................auauaauacaaccugcuaagugucc.................................................    5          0          seq
    .....................auauaauacaaccugcuaaguguccu................................................    7          0          seq
    .....................auauaauacaaccugcuaaguguccuag..............................................    1          0          seq
    .....................auauaauacaaccugcuaaguguccuagc.............................................    3          0          seq
    .....................auauaauacaaccugcuaaguguccuagcacu..........................................    1          0          seq
    ......................uauaauacaaccugcuaagu.....................................................    12         0          seq
    ......................uauaauacaaccugcuaagug....................................................    5          0          seq
    ......................uauaauacaaccugcuaagugu...................................................    76         0          seq
    ......................uauaauacaaccugcuaaguguc..................................................    14         0          seq
    ......................uauaauacaaccugcuaaguguccu................................................    2          0          seq
    .......................auaauacaaccugcuaagu.....................................................    5          0          seq
    .......................auaauacaaccugcuaagug....................................................    1          0          seq
    .......................auaauacaaccugcuaagugu...................................................    2          0          seq
    .......................auaauacaaccugcuaaguguc..................................................    4          0          seq
    .......................auaauacaaccugcuaagugucc.................................................    1          0          seq
    .......................auaauacaaccugcuaaguguccu................................................    2          0          seq
    ........................uaauacaaccugcuaagugu...................................................    1          0          seq
    ........................uaauacaaccugcuaaguguc..................................................    2          0          seq
    ........................uaauacaaccugcuaagugucc.................................................    5          0          seq
    ........................uaauacaaccugcuaaguguccu................................................    16         0          seq
    ........................uaauacaaccugcuaaguguccuagcacuuauca.....................................    1          0          seq
    .........................aauacaaccugcuaagugucc.................................................    1          0          seq
    .........................aauacaaccugcuaaguguccu................................................    4          0          seq
    .........................aauacaaccugcuaaguguccuagc.............................................    1          0          seq
    ..........................auacaaccugcuaaguguccu................................................    2          0          seq
```

oar-miR-374b

```
gaagaaauccuacucggauggauauaaauacaaccugcuaaguguccuagcacuuaucagguuguauuaucauuguccgugucuauggcucucguc
...........................auacaaccugcuaaguguccua...............................................	1	0	seq
...........................auacaaccugcuaaguguccuagc.............................................	1	0	seq
..................................................cacuuaucagguuguauuauc.........................	4	0	seq
...................................................Ccuuaucagguuguauuau..........................	1	1	seq
...................................................Ccuuaucagguuguauuauc.........................	1	1	seq
...................................................acuuaucagguuguauuauc.........................	16	0	seq
...................................................acuuaucagguuguauuauca........................	2	0	seq
...................................................acuuaucagguuguauuaucau.......................	17	0	seq
...................................................acuuaucagguuguauuaucauu......................	6	0	seq
....................................................cuuaucagguuguauuauc.........................	9	0	seq
....................................................cuuaucagguuguauuauA.........................	1	1	seq
....................................................cuuaucagguuguauuauca........................	8	0	seq
....................................................cuuaucagguuguauuaucau.......................	19	0	seq
....................................................cuuaucagguuguauuaucauu......................	522	0	seq
....................................................cuuaucagguuguauuaucauug.....................	3	0	seq
.....................................................uuaucagguuguauuauc.........................	1	0	seq
.....................................................uuaucagguuguauuaucau.......................	1	0	seq
.....................................................uuaucagguuguauuaucauu......................	77	0	seq
.....................................................uuaucagguuguauuaucauug.....................	1	0	seq
......................................................uaucagguuguauuaucauu......................	1	0	seq
```
